# Moving from information and collaboration to action: report from the 5th International Dog Health Workshop in Helsinki, June 2024

**DOI:** 10.1186/s40575-025-00143-0

**Published:** 2025-04-09

**Authors:** Katariina Mäki, Aimée Llewellyn-Zaidi, David St. Louis, Marc Ralsky, Dan G. O’Neill, Åke Hedhammar, Rowena M.A. Packer, Kari J. Ekenstedt, Jerold S. Bell, Becky Murphy, Ian J. Seath, Ambre Courtin, Mirkka Montonen, Anna Nygård, Vilma Reunanen

**Affiliations:** 1 International Partnership for Dogs, c/o Svenska Kennelklubben, Sollentuna, Sweden; 2 Pathobiology and Population Sciences, The Royal Veterinary College, Hatfield, Herts, United Kingdom; 3 Department of Clinical Sciences, Swedish University of Agricultural Sciences, Uppsala, Sweden; 4 Department of Basic Medical Sciences, College of Veterinary Medicine, Purdue University, West Lafayette, United States; 5 Department of Clinical Sciences, Cummings School of Veterinary Medicine, Tufts University, N. Grafton, MA United States; 6 TCI Veterinary Services, Feilding, New Zealand; 7 Dachshund Health, Kencot, United Kingdom; 8 Health and Genetic Resources Department, Société Centrale Canine, Aubervilliers, France; 9 Finnish Retriever Association, Turku, Finland; 10 Curly Coated Retriever Club of Finland, Turku, Finland; 11 Showlink Ltd, Espoo, Finland; 12 Department of Equine and Small Animal Medicine, University of Helsinki, Helsinki, Finland

**Keywords:** International Dog Health Workshop, International Partnership for Dogs, Dog health, Canine health and well-being, Canine genetics

## Abstract

**Background:**

The International Partnership for Dogs, together with a rotating national host organisation, holds approximately biennial meetings called the International Dog Health Workshop (IDHW). These workshops bring together a broad range of stakeholders in dog health and welfare, including scientists and veterinary practitioners, to improve the international sharing of information and resources, to provide a forum for ongoing collaboration, and to identify and agree on specific needs and actions to improve canine health and welfare.

**Workshop presentation:**

5th International Dog Health Workshop was hosted by the Finnish Kennel Club in Helsinki, Finland, in June 2024. The workshop was structured around four key issues facing those working to improve dog health: ‘Supply and Demand’, ‘Breeding for Health and Well-Being’, ‘Big Data’, and ‘Does the Colour Matter? Defining Breed vs. Variety’. The workshop provided an opportunity for participants to meet face-to-face after a five-year hiatus due to COVID-19, on the 10^th^ anniversary of the International Partnership for Dogs. Among the 106 decision-makers from 16 countries who attended the workshop, there was broad agreement on several issues during the discussions, such as following the scientific evidence on canine genetics and health, moving away from extreme conformation, and using all available tools, including crossbreeding, to maintain and increase genetic variation within dog breeds. It was agreed that these principles should become priorities for welfare-minded organisations at the national and international levels. Better education of puppy buyers, breeders, show judges, and other relevant parties was recurringly identified as a priority across all four themes of the workshop.

**Conclusions:**

In summary, key agreements from the 5th IDHW were that organisations must comply fully with relevant national animal welfare legislation, that organisations must work to eliminate extreme conformations from all dogs and to improve and maintain genetic diversity within subpopulations of dogs, and that organisations should recognise and support crossbreeding as an accepted and valuable tool for modern dog breeding.

## Abstract (es)

Breed-associated health problems in dogs are receiving increasing attention, and the last decade has seen legislative action in many countries against the breeding, acquisition, and promotion of dogs with traits that could lead to poor health, or highly inbred dogs. Much work is being done around the world to reduce preventable and predictable dog health issues, but the experience and knowledge available is often fragmented and discordant. Progress towards effective, improved welfare requires commitment to open collaboration among all those involved in dog health, science, and welfare. This report aims to outline the outcomes of a large meeting on dog health, The 5th International Dog Health Workshop, held in Finland in June 2024.

The International Dog Health Workshops (IDHWs) provide a forum to identify specific needs and actions aimed at improving the health, well-being, and welfare of dogs. The 5th workshop, attended by 106 decision makers from 16 countries, was structured around four key issues: ‘Supply and Demand’, ‘Breeding for Health and Well-Being’, ‘Big Data’, and ‘Does the Colour Matter? Defining Breed vs. Variety’.

The attendees represented a broad range of organisations, expertise, and geographic distribution. In addition to reinforcing established relationships, the intimate setting of the workshop provided the ideal environment to foster new connections among participants. These ongoing and new fruitful collaborations promote and enable sustainable action.

The 5th IDHW identified several priorities and new and ongoing actions that will be re-evaluated for implementation and impact at the next workshop in 2026. There was widespread agreement to follow the scientific evidence on canine genetics and health; to breed away from extreme conformation; to use all available tools, including crossbreeding, to maintain and increase genetic variation within breeds; and that organisations must comply fully with relevant national animal welfare legislation. It was agreed that these principles should become priorities for all welfare-minded organisations. It was noted that there is a need for better education of puppy buyers, breeders, show judges and other interested parties.

## Background

The International Partnership for Dogs (IPFD), in collaboration with a national host organisation in the respective land, conducts the International Dog Health Workshop (IDHW) at approximately biennial intervals. These workshops bring together experts and stakeholders from a variety of fields related to dog health, science, and welfare. The aim of these workshops is threefold: firstly, to facilitate global information exchange; secondly, to strengthen collaboration; and thirdly, to define concrete actions to enhance the well-being and welfare of all dogs.

The first IDHW was held in June 2012 in Stockholm, Sweden, where the need for an international platform for collaborative efforts was identified. This triggered the development of the IPFD, a nonprofit organisation initiated by several national kennel clubs and other stakeholders in dog health. The IPFD’s mission is to promote collaboration and the sharing of resources for the purpose of improving the health, well-being, and welfare of pedigree dogs and non-pedigree dogs around the world. In addition, the IPFD seeks to foster improved human-dog relationships [1].

DogWellNet.com was subsequently launched as the internet platform of the IPFD [2].

The second, third, and fourth IDHWs were organised by the IPFD together with a local host kennel club in Germany (2015), France (2017), and the UK (2019). From June 13th − 15th, 2024, the IPFD 5th IDHW was hosted by the Finnish Kennel Club in Helsinki, Finland, with Mars Petcare, Royal Canin, and Agria as major sponsors.

The 5th IDHW provided a forum to review the developments and outcomes during the first decade of IPFD, and then to identify future needs and actions to continue to support dog health and well-being.

An example of a successful initiative from the 3rd IDHW (2017) is IPFD’s Harmonization of Genetic Testing for Dogs (HGTD). Since its launch in 2018, the HGTD has expanded to include dynamic and transparent information on more than 80 international genetic test providers (GTPs), with more than 40 GTPs across 26 counties voluntarily providing detailed product and services data. This is a significant number of laboratories committed to openness and transparency. Transparent information improves consumer and health advisor confidence when selecting and recommending genetic testing providers. HGTD provides guidance on the appropriate selection and optimal use of genetic tests in dogs, based on currently available research and engagement with geneticists and expert advisors [3]. A related development, the Health Strategies Database for Dogs (HSDD), includes a comprehensive list of conditions (potentially inherited) by breed, country, and organisation or group for which there are developed management recommendations [4]. Combined, the HGTD and HSDD resources support health and breeding recommendations/counselling for breeders, owners, and veterinary professionals on genotypically and phenotypically assessed traits and diseases/conditions.

Another example of a successful outcome relates to the International Collaborative on Extreme Conformations in Dogs (ICECDogs), initiated following the 4th IDHW in Windsor, UK, in 2019. It is now leading international collaborative actions to eliminate extreme conformations from all dogs worldwide [5].

The 5th IDHW was structured around four key themes covering issues that regularly feature discussion points in relation to dog health: ‘Supply and Demand’, ‘Breeding for Health and Well-Being’, ‘Big Data’, and ‘Does the Colour Matter? Defining Breed vs. Variety’.

## Presentation of the meeting format and the themes

### Meeting format

The 5th IDHW was organised following a similar format to previous meetings, which has been detailed in the report published on the 3rd IDHW in 2017 [1].

In total, 106 participants from 16 countries attended the 5th IDHW, comprising decision leaders from most major stakeholder groups in dog health and welfare. The diverse list of attendees included breeders, members of breed club health committees, kennel clubs, breeding advisors, veterinarians, educators, researchers, geneticists, regulators, welfare organisations, industry, media, health campaigners, dog owners, and show judges, many of whom were able to offer experiences and insights across the dog world.

The 5th IDHW started on Thursday afternoon with presentations. Friday started with presentations from international experts in the morning, followed by breakout sessions for each theme in the afternoon. The workshop concluded with two sharing/summary sessions in plenum on Saturday morning.

The meeting went beyond mere discussion, with the aim of generating meaningful and concrete outcomes and agreed-upon action plans. Pre- and post-meeting resources and material for the 5th IDHW are available on DogWellNet.com [6]. This paper summarises the discussions, recommendations, and actions identified and committed to by participants during the workshop.

### ‘Supply and Demand’ and ‘Breeding for Health and Well-Being’

The COVID-19 pandemic increased the demand for dogs and other pets, leading to an increase in the number of puppies acquired in several countries. In the UK, the People’s Dispensary for Sick Animals (PDSA) ‘Animal Wellbeing (PAW) Report 2022’ estimated that 2.5 million dogs had been acquired since the start of the pandemic in 2020 [7].

In addition to the increased volume of puppy sales, puppy buying behaviours deteriorated from 2020 to 21, including puppies being less likely to be seen with their mothers and being collected away from where they were bred, increasing the likelihood of buyers supporting poor welfare suppliers and illegal imports [8, 9].

The ‘pandemic puppy’ boom was swiftly followed by escalating prices of veterinary care, basic dog husbandry, and feeding due to the cost-of-living crisis, meaning that many owners are faced with the dilemma that they cannot afford to keep their dog [10, 11]. In addition to the increased financial cost of ownership, higher levels of behavioural problems due to a lack of socialisation during the pandemic have been documented in this pandemic generation of dogs, leading to some owners relinquishing their dogs [12–14]. This has added to the already high number of unwanted dogs in the world.

Also, illegal suppliers commonly operate in this area. For example, the illegal puppy trade in Europe has been characterised as a highly organised, buoyant, and lucrative enterprise, exploiting many well-meaning consumers into purchasing puppies reared under poor welfare conditions but sold under staged conditions by third parties [15].

Furthermore, international groups may deliberately produce ‘homeless’ dogs in puppy mills for ‘rescue’ by compassionate but often misguided people. Better legislation with stronger enforcement is needed to eliminate illegal activities and protect the welfare of both puppies and breeding dogs [16]. New animal welfare legislation and/or new enforcement criteria have recently come into force in many countries, such as Germany and the Netherlands [17, 18].

In addition to concerns that basic welfare needs in early life are often not met in the aforementioned supply systems, there is a growing concern about effective protection of dog health among dog enthusiasts and the public regarding specific aesthetic characteristics that are in demand but associated with poor welfare. Due to such trends, dog breeding often does not meet all the requirements of relevant legislation (where present), for example, in terms of dogs being bred to be capable of normal reproduction, free from otherwise preventable disease and able to express basic behavioural needs. Many physical breed characteristics, e.g., entropion/ectropion, skin folds, and extreme brachycephaly, are considered health risks [5]. Extreme conformation, as preventable and purposeful selection-driven features that damage innate health are perhaps the most pressing welfare issue for domestic dogs at present [5].

ICECDogs has defined extreme conformation in dogs as “*a physical appearance that has been so significantly altered by humankind away from the ancestral natural canine appearance that affected dogs commonly suffer from poor health and welfare*,* with negative impacts on their quality and quantity of life*” [19].

There is a growing body of data and information on the health problems caused by extreme conformations, but this evidence may not be used as effectively as it should be to eliminate these traits from dogs. One of the main barriers to progress here is that many owners and breeders fail to recognise health problems, discomfort, and pain in their dogs related to extreme conformation but instead normalise these as being ‘normal for breed’ [20–22].

Every dog should be free to enjoy a life with good ‘innate health’, and animal welfare legislation should aim to make this more of a reality, but limited or inconsistent enforcement is hampering welfare progress [23], and enacted laws do not always fulfil their function of protecting dogs against these harmful traits. Although surveys of public opinion often report that health and welfare of dogs are the most important factors for the future [24], the evidence on actual puppy purchasing decisions often do not reflect this, e.g., growing international trends towards extreme brachycephalic and chondrodysplastic dogs. As such, legislation is likely needed to prevent these harms and remove these choices from consumers, who may not be aware of the serious welfare impacts of their decisions.

Improved animal welfare legislation for breeding is certainly needed, but the interpretation and enforcement of current, new, and updated legislation is often vague or inconsistent. In Germany, for example, the specific rules vary from state to state, although the law (Animal Welfare Regulation for the Protection of Dogs) is the same throughout the country [25]. Clear legislation and enforcement must be based on scientific evidence and should be as precise and consistent as possible to ensure people are able to understand and comply with the law. Without thoughtful and evidence-based laws and guidance in place, interpretation of laws will create uncertainty for control authorities, veterinarians, event organisers, and dog owners, as has happened in Germany. The German Kennel Club has stated that the lack of clarity, and inconsistent interpretation of the law results in disproportionate orders that violate animal welfare [26].

Good-faith and regular cooperation between governments, scientists, and kennel clubs is therefore needed for optimal legislation and enforcement.

### Big Data

Phenotypic and genomic data on canine health, longevity, and inbreeding/genetic diversity have been collected by various organisations over recent decades. Almost all kennel clubs hold extensive records on testing for hip and elbow dysplasia, eye diseases, and other diseases. In addition, large volumes of data exist as clinical notes in veterinary clinic electronic record systems. The big question is how to support effective research efforts to curate, explore, and report these data in formats that can be used by kennel clubs, breeders, and the public to breed and purchase healthier dogs. ‘Big Data’ offers endless opportunities, but realising these opportunities requires long-term investment in data analysis projects and personnel to develop the methods needed to harness the power of these data.

Country-specific big data are being used to develop estimated breeding values (EBVs) for hip and elbow dysplasia, spinal problems, and/or behavioural traits by the relevant kennel clubs in Finland, Denmark, Norway, Sweden, and the UK. EBVs can be made more accurate when good quality data from different countries are combined and include genomic information, as is now commonplace in the livestock industry [27]. The use of international, aggregated big data poses various challenges in terms of data storage, ownership, transfer, and analysis. Although accurate EBVs offer potential for efficient selection away from certain disorders, this also presents considerable challenges for managing genetic diversity in subpopulations, which may already be highly compromised.

### Does the Colour Matter? Defining Breed vs. Variety

One commonly proposed option to reduce the frequency and severity of disorder occurrence in dog breeds is to identify and selectively breed from healthier dogs and lines within breeds. However, all forms of selection can reduce genetic diversity [28–30]. In the Netherlands, Sweden, and Finland, breed clubs have worked with kennel clubs to increase genetic diversity in national breeds by, for example, routinely allowing exemption registrations of breed-typical individuals to ensure long-term sustainable breeding [31, 32].

By considering closely related current breeds to be varieties of the same parent breed, crossbreeding between these varieties could improve genetic diversity. In Sweden, this type of crossbreeding is promoted in numerically small Swedish hunting dog breeds. The aim is to increase genetic variation and to maintain and improve health and hunting traits. The donor breeds have similar hunting preferences and appearances [33].

A successful proof-of-concept for modern pedigree dog breeding came in 2006, when the Finnish Spitz and its Russian sister breed, the Karelo-Finnish Laika, were declared to be the same breed. This broadened the gene pool of the Finnish Spitz and allowed for a valuable increase in genetic variation [34].

Crossbreeding, i.e., the introduction of genetic variation by mating with another distinct breed (donor breed), can be used as a tool to address specific physical or genetic health issues in individual breeds after much thought and planning. To date, this has been done in only a few of the many breeds that are struggling with serious health issues. One example is the Cavalier King Charles Spaniel, for which Finland, Norway, and Sweden have all started such a project [35–37]. The aim is to increase genetic variation and improve health. The chosen donor breeds are not closely related, are free of the typical hereditary diseases of the Cavalier, show appropriate behaviour for a companion dog, and are of similar size. The shape of the skull is also considered in donor breeds, as extreme changes in skull morphology are risk factors for many health problems, including syringomyelia [38].

It must be acknowledged that most current pedigree dog breeds were originally created through crossbreeding [39, 40]. Crossbreeding is not a new tool, but rather a proven method of breeding towards health and away from disease in dogs. Knowledge and acceptance of how breeds have evolved in the past and how breeds relate to each other are essential when planning to breed healthier dogs; most pedigree breeds were originally crossbred. It is also necessary to shape opinions and attitudes away from strict adherence to “pure” breeds and instead move towards an understanding of biological facts and the need for genetic diversity while maintaining innately healthy breed types and temperament [41].

## Discussion and conclusions

The discussions that took place, and the actions proposed and subsequently agreed to by participants for each theme are summarised below and in Table 1.

**Table 1:** Identified priorities and planned actions in the 5th International Dog Health Workshop

Theme; Leader	Actions/statements
Supply and Demand. Peter Friedrich, Germany	Illegal activities such as illegal importation need to be addressed through more effective legislation and enforcement. Explore new opportunities for better collaboration between researchers and other interested parties with greater access to consumers and breeders to provide the data and information on dog buyers needed to guide action. Meet virtually on a regular basis, to ensure continued collaboration and exchange of ideas.
Breeding for Health and Well-Being. Kirsi Sainio, Finland	It is imperative that the well-being and lives of dogs are prioritised in all canine activities. Discussions need to continue, but the solutions improving canine health and well-being need to be found urgently. Kennel clubs need to communicate better and more forcefully with their judges to make them understand their role and responsibility in protecting the health and well-being of all breeds. More effective education and understanding within puppy buyers, breeders, and show judges is needed to ensure research and evidence are put at the heart of the action. All stakeholders who care about dogs should strive to be champions of canine health and well-being first and then to consider how this relates to maintaining existing breed concepts.
Big Data. Kari Ekenstedt, USA	Launch a pilot project to test data collection and analysis. Set up a working group to identify which traits have currently Estimated Breeding Values (EBVs) in different countries and how these EBVs can be used more effectively. Create an online resource on DogWellNet.com to list key datasets held by different organisations for research purposes.
Does the Colour Matter? Defining Breed vs. Variety. Heidi Parker, USA	Routine crossbreeding between varieties of the same breed should be allowed and even promoted. Move towards harmonisation of rules and terminology regarding breed varieties and crossbreeding. Gather information about the rules on crossbreeding in different countries. Make a series of webinars available globally through DogWellNet.com covering the history of breeding and experiences with crossbreeding, including best practices and common pitfalls. All stakeholders should give more attention to work to increase genetic diversity in dog breeds.
All themes	Compile a list of studies related to the four workshop themes to be shared on DogWellNet.com. Educate puppy buyers, breeders, show judges, and other interested parties on issues that affect canine health and welfare.

### Supply and Demand

In this context, illegal activities such as illegal importation need to be addressed through more effective legislation and enforcement. Dogs, sellers, and owners should be identifiable and traceable. This could be accomplished through government registers, i.e., compulsory identification of all dogs and registers of owners and sellers. The forthcoming new European Union (EU) legislation on the welfare of dogs and cats and their traceability will make compatible registers compulsory across the EU [42].

Anyone considering acquiring a dog should be directed towards a responsible supply that complies with national animal welfare legislation. All the participants present committed to directing puppy and adult dog buyers towards such supply. One step in this direction could be the development of international certification for responsible breeding. It was agreed that this would improve the image of and access to responsible breeders, which is currently extremely challenging for buyers.

The question of how a prospective dog buyer can identify and recognise a responsible operator was explored. Basic criteria should be developed to define high-welfare breeding practices.

A clear public message agreed to be extremely important was that a dog is not an inanimate consumer product but is a sentient animal that is often a family member that can live up to 15 years and that, along with all the joy and happiness they bring, there is effort and cost involved for the owner.

It was discussed that many people seeking a dog often must wait months to acquire a higher-welfare puppy, which can increase the temptation to buy a more readily available puppy from an unclear background. However, a responsible buyer will be prepared to wait for the right dog from the right background. It was considered important to highlight to the public, for example, how long it takes to responsibly breed a dog and the extra value and safeguards that come with acquiring a responsibly bred dog without an extreme conformation or potential health issues, and the value of good-quality puppy socialisation.

Better messaging to promote value to owners from purchasing only from responsible breeders was agreed upon. For example, messaging should highlight the reduced maintenance costs of acquiring an innately healthy dog compared with an unhealthy dog, e.g., not acquiring a dog with undesirable conformation, or discourage buying from a seller that may be able to provide a puppy ‘instantly’ but will not show the puppy with their mother, which is now known to be associated with poorer health [43] and worse behavioural outcomes [44].

It was agreed that this message should be shared as consistently as possible from various stakeholders, such as the veterinary profession, kennel clubs, breeders, animal welfare organisations, and animal shelters.

More research was considered to be required into supply and demand trends, such as which breeds are most popular at any given time in any country or region, and ‘early warning’ systems for spikes in breed popularity that can lead to illegal or unethical puppy producers cashing in on this demand. Campaigns to increase demand for currently less popular breeds that have good innate health potential could create more diversity across the dog population. The excessively high popularity of any single breed tends to create a questionable supply and may promote breeding, which can ultimately lead to greater welfare issues.

Understanding the mechanisms of illegal trade was acknowledged as an important step in tackling this problem. Options for gathering information about illegal trade and supply were explored. Obtaining information directly from owners may be difficult because owners are often sold a puppy without reliable evidence on its provenance due to the sophisticated tactics used by the illegal puppy trade to obscure this.

The group agreed to explore new opportunities for better collaboration between researchers and other interested parties, with greater access to consumers and breeders to provide the data and information on dog buyers needed to guide action.

The group planned to meet virtually on a regular basis, hosted by the IPFD. This will ensure continued collaboration and exchange of ideas between interested parties.

### Breeding for Health and Well-Being

Many difficult and challenging questions about breeding for health and well-being were posed during the discussions. Do human beings hold the social licence to continue our current practices, which have been in place for the last 200 years? Is there still time to act for better health, or is it already too late, especially for some pedigree dog breeds? How can better health be achieved? What to do about breeds who are notable for their extreme conformation? Can such breeds continue to be bred, without extremes of conformation, and if so, should they? Have kennel clubs already lost their social licence, and if so, how can they get it back? Are the new laws moving in the right direction?

Is everyone ready to accept the need to change and eliminate extreme characteristics? A few participants noted that some breed enthusiasts and breed clubs want more evidence about the state of their breed.

It was agreed that the fact is that breeds are changing (and always have), and we should manage that change for better health. Breed Watch, an online resource from the UK Kennel Club, tracks trends toward more extreme conformations and advises against unhealthy trends [45]. It raises awareness of specific concerns; helps show judges identify and reward dogs with less extreme conformations; and collects data to monitor and address visible health issues. A similar monitoring tool, Breed-Specific Instructions, is used in the Nordic and German kennel clubs [46, 47].

It was acknowledged that discussion about health issues within breeds sometimes made people defensive, which consequently makes it difficult for different stakeholders to work together. Instead of breeds, it is often more useful and inclusive to talk about harmful traits (e.g., extreme conformation) that can develop in any breed and that everyone can agree should be discouraged in any dog.

The broader question was posed as to why extreme traits have been developed in dogs in the first place. One suggestion was because of the breed standard. It was agreed that any breed standard containing problematic wording that leaves too much room for interpretation and encourages the development of extreme traits should be changed. It was agreed that humans have devised breed standards, and therefore, humans can change them. There was also a sentiment expressed that the problem is often not the breed standards themselves, but rather their misinterpretation by judges and breeders, or even a view that very few of these people may have even read the relevant breed standards.

Judges were considered to play a key role in the direction of the show segment of each breed, so judge education is very important. It was agreed that kennel clubs need to communicate better and more forcefully with their judges to help them understand their role and responsibility in protecting the health and well-being of all breeds.

However, not only judges but also breeders and the public need to be better educated on how to protect the health of dogs. It was agreed that each breed should have a breeding programme that identifies extreme conformation risks and provides practical and clear breeding guidelines to reduce and avoid these risks. The UK Kennel Club has veterinary checks at championship shows for breeds identified as having health problems associated with exaggerated features [45]. This was applauded as a good step forward.

The influence of kennel clubs on overall dog health is dual. In most countries, most dogs are bred and owned outside of kennel clubs. Animal welfare legislation applies to all dogs, so its implementation and enforcement should affect all breeders and sellers, not just those who register their puppies with kennel clubs. In England, animal welfare legislation expressly states that ‘…*no dog may be kept for breeding if it can reasonably be expected*,* on the basis of its genotype*,* phenotype or state of health that breeding from it could have a detrimental effect on its health or welfare or the health or welfare of its offspring’* [48]. However, kennel clubs do have an influence and responsibility beyond the dogs they register by setting the standard for breeds and for “lookalikes”– dogs of a particular breed without kennel club registration.

To ensure that all breeders comply with their legal requirement to never breed from dogs with extreme conformation phenotypes (e.g., extreme brachycephaly, excessive skin folds), it was agreed that all breeders/producers should be registered, and that kennel clubs may be suitable for creating registers for all dogs regardless of pedigree status. If known, the pedigrees of previously unregistered dogs could also be recorded.

The Orthopedic Foundation for Animals (OFA) also gives disease test results for hybrid dogs [49]. It was agreed that test results of these dogs would benefit the health of all dogs if they were included in the kennel club databases.

Productive and regular cooperation between governments, scientists, and kennel clubs was seen as essential to creating enforceable laws that truly improve dog health. This dialogue should be two-way, whereby it was also considered important to inform the government about what is already being done. For example, the UK Kennel Club’s ‘Play your Part - Breeding, Buying and Bringing Up Brachycephalic Dogs Better’ is an example of an informative public approach that promotes the UK Brachycephalic Working Group public message to ‘Stop and think before buying a flat-faced dog’ [50, 51].

It is important for breeders to know what information is available and how to use it to improve their breeding outcomes. Some delegates pointed out that, currently, there is so much information available that it can be difficult to keep track of it and sometimes difficult to interpret the clinical significance and usefulness of the information.

Some European kennel clubs have DNA working groups that review different DNA tests and offer help to breeders. In addition, the IPFD HGTD database is a unique, free portal for information on genetic testing laboratories, genetic tests, and tests by breed, and for all dog types [3].

There was wide agreement that, universally, debate and agreement about what needs to be done is often challenging because countries differ from each other in legal, cultural, welfare, socioeconomic, and other ways. In Europe and the EU, for example, prioritising canine health is considered more advanced than in some other parts of the world. There was a view that the Fédération Cynologique Internationale (FCI) [52] could play a role as a clearing house and bridge between countries; however, unfortunately, the FCI was not formally represented at the workshop (although many attending people had “multiple hats” [53], including several FCI show judges). The assembled delegates were optimistic that the FCI will still step up and play some role in wider collaborative efforts in the future.

The solutions proposed during this theme focused on more effective education and understanding among puppy buyers, breeders, and show judges, putting research and evidence at the heart of the action.

Compliance with existing national and international legislation was agreed upon as essential, particularly for breeds that cannot be legally bred under current conditions. It was agreed that necessary phenotypic or genotypic changes should be made for breeds having or at risk for extreme conformations but that this requires consensus on the need for– and approach to– these changes.

It was agreed that animal welfare legislation can act as a lever for positive change, with greater social pressure resulting in actual change. Social pressure as an effective tool should be included as part of any strategy for behavioural change. In dog breeding, this would work by making it socially unacceptable to produce, promote, sell, or buy dogs with a known, high risk of disease. At the same time, rewarding good practices can help raise awareness and encourage good behaviour. It was agreed that the most effective audience for attitudinal education is young people, who are future breeders and owners [54].

Sharing information about successful breeding programmes encourages other interested parties to improve their practices. For this purpose, the IPFD has a database where information can be entered (Health Strategies Database for Dogs, HSDD) [4].

It was agreed that, although the discussions need to continue, solutions need to be found urgently. Despite the diversity of legislation, cultures, and societies, dogs remain consistent and universal in their biological nature and needs. The welfare and life of dogs should be prioritised in all canine activities. It was agreed that all stakeholders who care about dogs should strive to be champions of dog health and welfare first and then consider how this relates to the preservation of existing breed concepts.

### Big Data

To make the best use of existing veterinary data, machine learning and artificial intelligence could be used to collect important clinical data from existing electronic medical records for breeding purposes.

Estimated Breeding Values (EBVs) are powerful tools, but their implementation requires big data. However, many dog breeds include very few numbers of registered dogs and therefore have ‘small data’. For these breeds, data from several sources could be combined to enhance the EBVs.

One possibility proposed involved international disease registries that actively rather than passively collect health data. This raised many questions about who would be responsible for maintaining the registries and who would provide the data on phenotypes for different diseases– this could be either the dog owner or the veterinarian or some other evidence provider. Other challenges included whether the diagnoses would need to be verified by a specialist, how would information on the health status of dogs be kept up to date as it changes over time, and who would own the data.

Another challenge identified was the lack of assessment standardisation for many areas related to canine health. Screening tests for the same disease can differ across countries and organisations, so the data produced may not be comparable, e.g., different hip dysplasia grading systems by the BVA [55] and the FCI [56]. The diagnostic codes used in veterinary clinical programs vary, with some, for example, being based on VeNom coding [57] or Pyramidion [58]. The methods of microchip identification for dogs are not standardised across countries.

If the differences between the various data collection systems are known, it becomes possible to take them into account in statistical analyses. It was agreed that it takes much time– and money– to convert large amounts of data into an analysable format and then provide basic information and tools for breeding.

In addition to the aforementioned challenges, privacy-related legislation, such as the General Data Protection Regulation (GDPR) in Europe [59] must be considered. To use a dog’s test results as part of a large dataset, permission ideally should be sought from the owner. The participants suggested the creation of a standardised form to document the consent of the dog’s owner. The form could be used nationally and internationally for different studies and for breeding value estimation.

Existing international dog breed databases, human biobanks, and data systems and technologies related to livestock could provide inspiration and risk identification to support new canine Big Data project development.

To make the best use of big data, collaboration is needed between kennel clubs themselves, and research organisations. The participants decided to launch a pilot project to test data collection and analysis, ideally in a small breed with regular gene flow between countries.

The participants also set up a working group to identify which traits currently have EBVs in different countries and how these EBVs can be used more effectively. The working group also aimed to produce training materials on the use of EBVs in breeding. If breeders do not trust EBVs, there is little point in spending time and money developing them and linking them to genomic data.

Further education is also needed in the understanding and use of inbreeding coefficients estimated by different methods and in the understanding and application of genetic testing; test results may not always provide useful interpretation and application information.

The participants also discussed maintaining a dedicated web page on DogWellNet.com to collect and annotate scientific papers on specific topics. This resource would include lay summaries that provide accessible scientific evidence to support better use of various breeding tools, such as EBVs. This site would be a valuable resource for breeders and breeding advisors, providing scientific support for their advice and decisions.

In addition, participants suggested the creation of an online resource on DogWellNet.com, like the ‘Genetic Testing Providers’ (GTP) database [60]. The new resource would list key datasets held by different organisations. It would show what kind of datasets are available and who to contact for accessing data. This information would be of great interest to researchers and others in the big data field. Examples of currently available big data include the Golden Retriever Lifetime Study [61] and VetCompass [62]. These datasets are openly available to all researchers, and the research findings are often applicable to other dogs as well as humans.

### Does the Colour Matter? Defining Breed vs. Variety

The group discussed ways to increase genetic diversity in dog breeds. Exploring all available means to increase diversity was considered important by the participants. Suitable breeding dogs of the same breed can often be found outside official registries. There are many excellent and healthy purebred dogs worldwide that are not registered in their respective kennel clubs, and these dogs could be used in breeding programmes.

It was agreed that it would be sensible to allow and even promote routine crossbreeding between varieties of the same breed. The FCI accepts this [63], but many breed clubs have stricter rules that prevent this form of crossbreeding. The group discussed the definition of a breed variety, which varies from country to country. For this, as for crossbreeding, it is important to move towards harmonisation of rules and terminology.

The participants decided to have a questionnaire sent to representatives of different countries to gather information about their rules on crossbreeding [64, 65]. A summary will be published on DogWellNet.com. The next IDHW can then focus on harmonising the rules.

The participants discussed the issues that make crossbreeding difficult for many to accept, or even talk about, and explored the pros and cons of owning a crossbred dog, i.e., why some people actively want to own a crossbred dog. It was considered that crossbred dogs born within a kennel club-approved project should be recognised as belonging to a particular breed and should have access to all the same hobbies and competitions as “purebreds”.

Consistent with the other themes, this group recognised a strong need for better communication. This could function to update people’s perceptions of crossbreeding and even explore why people think about individual breeds or about purebred vs. crossbred dogs in the way we do. There was a view that even if/when crossbreeding is allowed and accepted as a standard option for better dog breeding, this would not automatically mean that every breeder would choose to use crossbreeding. There was agreement that it must be clearly stated that the purpose of crossbreeding for kennel club projects is to reduce specified health issues in the current breed by, for example, widening genetic diversity or moving towards better innate health. This was agreed to be a very different purpose than creating new breeds [66]. It was noted that the public communication should also emphasise that diversity will not be restored with one or two crossed litters, but that ongoing crossbreeding will be needed on a regular and continued basis.

Open-mindedness must be encouraged by educating breeders and breed societies about the true history of dog breeding. Breed registries used to be more open. It was considered valuable to include easy-to-understand articles in breed club magazines explaining why, how, and when to crossbreed. Sharing pictures of dogs from crossbreeding programmes that are typical of the breed was also seen as helping to allay fears about crossbreeding.

The participants suggested that a series of webinars could be made available globally through DogWellNet.com, covering the history of breeding and experiences with crossbreeding, including best practices and common pitfalls.

It was agreed that more attention should be given to work being done to increase phenotypic and genetic diversity. The UK Kennel Club was encouraged to explore the possibility of an international crossbreeding project exhibition at Crufts [67] and to consider an international dog health award for crossbreeding projects.

### Plenary discussion

When all participants gathered again on the final day of the 5th IDHW, discussions expanded to the need to reach out to new partner organisations and people, the need for better awareness and education, and increased sharing of information on the IPFD site, DogWellNet.com.

There was overall agreement that there is still a wide divide in the relative focus on, and promotion of, dog health across the various organisations involved with dogs. It was agreed that all interested parties must actively work to be as inclusive as possible and to reduce differences between groups.

It was widely agreed that stakeholders in canine health and welfare have already achieved many positive changes over the past decade but that we need to become more inclusive and to include and be concerned about all dogs, not just the pedigree subset of dogs. The goal of IPFD since the first IDHW in 2012 has been to bring all parties to the table, and this inclusivity has been steadily and successfully improving over time, with an increasing number of countries taking part in each workshop. However, despite the current diversity of participants, there were still some groups absent or poorly represented that could strengthen future discussions, such as the legislators, of which only a few were present, and the FCI.

A common challenge identified across all four themes was how best to engage more people in good education. Education and communication were considered critical for encouraging positive changes in human behaviour; therefore, reaching a larger proportion of puppy buyers and unorganised breeders is important. Although most dogs in Nordic countries are on the pedigree register, this is not the case elsewhere where many dogs are not on any formal register, and this limits direct access to information sharing with owners and breeders. This leaves a challenge for sharing health promotion work and breeding advice with owners and breeders of dogs that do not appear on any formal or informal register.

In addition to the workplans discussed above, it was also agreed that a list of studies related to the four workshop themes would be compiled and shared on DogWellNet. When the DogWellNet.com website was created in 2014, it comprised just one page. In the ten years since then, this information hub has grown to include nearly 1000 pages where it can now be difficult to find specific information. It was announced that the site is currently being redeveloped to streamline navigation.

The active participation of different actors was explained as being very important for the maintenance and growth of DogWellNet.com [2]. DogWellNet is an information exchange platform managed by the IPFD, but the IPFD itself does not have the resources to search for and upload all the information available, so it is up to all stakeholders to actively submit. For example, the completion and maintenance of the HSDD database requires the active involvement of kennel and breed clubs in different countries. It was discussed that it would be useful to share information about successes on DogWellNet and that this could then act as a good starting point for building new successes.

## Conclusions

Key agreements from the 5th IDHW 2024 were that all organisations must comply fully with relevant national animal welfare legislation, that all organisations must work to eliminate extreme conformations from all dogs and to increase genetic diversity within subpopulations of dogs, and that all organisations should embrace crossbreeding as an accepted and valuable tool for modern dog breeding.

Progress towards achieving the action plans specified within each theme at the 5th IDHW will be presented and reviewed at the 6th IDHW, tentatively scheduled to take place in conjunction with the 2026 World Dog Show and FCI’s International Judge’s Congress in Bologna, Italy. New stakeholder groups and delegates will be encouraged to attend the 6^th^ IDHW to further broaden the perspectives.

## Data Availability

No datasets were generated or analysed during the current study.

## Declarations

### Disclaimer

International Partnership for Dogs is grateful for the thoughtful, informed input of the 5th International Dog Health Workshop (IDHW) participants, who represent a broad cross section of our global canine community. The views, discussions, and conclusions captured in this report are those of the workshop participants and do not necessarily reflect the opinions or positions of the International Partnership for Dogs (IPFD), Suomen Kennelliitto (The Finnish Kennel Club), or the report’s authors. The publication of this report is intended to facilitate further discussion of the challenges and opportunities addressed within the four workshop themes. IPFD is leading these ongoing discussions in which the participants will evaluate recommendations, establish priorities, determine resource requirements/availability, and formulate plans for collaborative actions– while ensuring alignment with the Mission, Vision, Values, and Goals of the IPFD [68].

## Abbreviations

1st IDHW: 1st International Dog Health Workshop 2012
2nd IDHW: 2nd International Dog Health Workshop 2015
3rd IDHW: 3rd International Dog Health Workshop 2017
4th IDHW: 4th International Dog Health Workshop 2019
5th IDHW: 5th International Dog Health Workshop 2024
6th IDHW: 6th International Dog Health Workshop 2026
AKC: American Kennel Club
BOAS: Brachycephalic obstructive airway syndrome
FCI: Fédération Cynologique Internationale
GTP: Genetic test provider
HGTD: Harmonization of Genetic Testing for Dogs
HSDD: Health Strategies Database for Dogs
IPFD: International Partnership for Dogs
